# Clinical Profile and Determinants of Mortality in Patients with Interstitial Lung Disease Admitted for COVID-19

**DOI:** 10.3390/jcm12113821

**Published:** 2023-06-02

**Authors:** Alba Mulet, Iván J. Núñez-Gil, Juan Antonio Carbonell, Joan B. Soriano, María C. Viana-Llamas, Sergio Raposeiras-Roubin, Rodolfo Romero, Emilio Alfonso-Rodríguez, Aitor Uribarri, Gisela Feltes, Victor M. Becerra-Muñoz, Francesco Santoro, Martino Pepe, Alex F. Castro-Mejía, David Chipayo, Miguel Corbi-Pascual, Javier López-Pais, Oscar Vedia, Edoardo Manzone, Germán Molina-Romera, Carolina Espejo-Paeres, Álvaro López-Masjuan, Lazar Velicki, Antonio Fernández-Ortiz, Ibrahim El-Battrawy, Jaime Signes-Costa, on behalf of HOPE Team

**Affiliations:** 1 Pulmonary Department, Hospital Clínico Universitario Valencia, INCLIVA, 46010 Valencia, Spain; 2 Cardiology Department, Hospital Clínico San Carlos, Instituto de Investigación Sanitaria del Hospital Clínico San Carlos (IdISSC), 28040 Madrid, Spain; 3 Bioinformatics and Biostatistics Unit, INCLIVA, 46010 Valencia, Spain; 4 Faculty of Medicine, Universitat de les Illes Balears, 07120 Palma, Spain; 5 Centro de Investigación en Red de Enfermedades Respiratorias, Instituto de Salud Carlos III, 28029 Madrid, Spain; 6 Intensive Medicine Department, Hospital Universitario Guadalajara, 19002 Guadalajara, Spain; 7 National Center for Cardiovascular Research (CNIC), Department of Cardiology, Álvaro Cunqueiro University Hospital, 36312 Vigo, Spain; 8 Emergency Department, Hospital Universitario Getafe, 28905 Madrid, Spain; 9 Bellvitge University Hospital, 08907 Barcelona, Spain; 10 Cardiology Department, Hospital Clínico Universitario Vall D’Hebrón, 08035 Barcelona, Spain; 11 Cardiology Department, Hospital Universitario Vithas Arturo Soria, 28043 Madrid, Spain; 12 Cardiology Department, Hospital Clínico Universitario Virgen de la Victoria, 29010 Malaga, Spain; 13 Department of Medical and Surgical Sciences, University of Foggia, 71122 Foggia, Italy; 14 Cardiology Department, Azienda Ospedaliero-Universitaria Consorziale Policlinico di Bari, 70124 Bari, Italy; 15 Hospital General del Norte de Guayaquil IESS Los Ceibos, Guayaquil 090615, Ecuador; 16 Department of Cardiology, Hospital Universitario de Cáceres, 10004 Cáceres, Spain; 17 Department of Cardiology, Complejo Hospitalario Universitario de Albacete, 02008 Albacete, Spain; 18 Department of Cardiology, Complejo Hospitalario Universitario de Ourense, 32005 Ourense, Spain; 19 Instituto de Investigación, Sanitaria del Hospital Clínico San Carlos (IdISSC), Hospital Clínico San Carlos, Universidad Complutense de Madrid, 28040 Madrid, Spain; 20 Hospital del Sureste, 28500 Madrid, Spain; 21 Department of Preventive Medicine, Hospital Santiago de Compostela, Instituto de Investigaciones Sanitarias de Santiago de Compostela, 15706 A Coruña, Spain; 22 Hospital Príncipe Asturias, 28805 Alcalá de Henares, Spain; 23 Department of Cardiology, Hospital Universitario Juan Ramon Jimenez, 21005 Huelva, Spain; 24 Faculty of Medicine, University of Novi Sad, 21000 Novi Sad, Serbia; 25 Institute of Cardiovascular Diseases Vojvodina, 21204 Sremska Kamenica, Serbia; 26 Department of Cardiology, University Medical Center Mannheim, 68167 Mannheim, Germany

**Keywords:** interstitial lung diseases, COVID-19, mortality, ventilatory support

## Abstract

Background: Concern has risen about the effects of COVID-19 in interstitial lung disease (ILD) patients. The aim of our study was to determine clinical characteristics and prognostic factors of ILD patients admitted for COVID-19. Methods: Ancillary analysis of an international, multicenter COVID-19 registry (HOPE: Health Outcome Predictive Evaluation) was performed. The subgroup of ILD patients was selected and compared with the rest of the cohort. Results: A total of 114 patients with ILDs were evaluated. Mean ± SD age was 72.4 ± 13.6 years, and 65.8% were men. ILD patients were older, had more comorbidities, received more home oxygen therapy and more frequently had respiratory failure upon admission than non-ILD patients (all *p* < 0.05). In laboratory findings, ILD patients more frequently had elevated LDH, C-reactive protein, and D-dimer levels (all *p* < 0.05). A multivariate analysis showed that chronic kidney disease and respiratory insufficiency on admission were predictors of ventilatory support, and that older age, kidney disease and elevated LDH were predictors of death. Conclusions: Our data show that ILD patients admitted for COVID-19 are older, have more comorbidities, more frequently require ventilatory support and have higher mortality than those without ILDs. Older age, kidney disease and LDH were independent predictors of mortality in this population.

## 1. Introduction

The COVID-19 pandemic has affected more than 600 million people around the word, and more than 6 million deaths have been counted to date, according to Johns Hopkins University data [1].

The clinical presentation of COVID-19 has a wide spectrum that ranges from asymptomatic or paucisymptomatic patients to the development of bilateral pneumonia, respiratory failure, respiratory distress and death [2]. The severity of COVID-19’s involvement varies according to the underlying diseases of the patients, among other factors [3].

Interstitial lung diseases (ILDs) are a heterogeneous group of diffuse parenchymal lung disorders with known or unknown causes that lead to inflammation and fibrosis of the alveoli, distal airways and septal interstitium of the lungs [4,5,6,7]. Within the clinical course of an ILD, acute exacerbation can occur at any time, and it is associated with significant morbidity and mortality [8,9,10].

Some studies have shown that the proportion of patients with an ILD is higher among those infected with COVID-19 than in the general population, suggesting that they have a higher susceptibility to infection [11,12]. In addition, patients with ILDs already have impaired lung function, and viral infections are a risk of exacerbation for them [13,14]; thus, it is expected that COVID-19 will have a more severe impact on them.

The aim of this study was to characterize ILD patients admitted for COVID-19 and to determine factors that are associated with a worse prognosis, including either ventilatory support or death.

## 2. Materials and Methods

### 2.1. Study Design and Population

Data were obtained from the HOPE (Health Outcome Predictive Evaluation) COVID-19 registry (https://hopeprojectmd.com (accessed on 6 Abril 2020)) on ClinicalTrials.gov (NCT04334291), a multicenter international registry. Patients with ILDs were identified from the cohort. Demographic and clinical data at baseline, upon admission and in-hospital were analyzed.

In brief, the HOPE COVID-19 registry is an international initiative designed as a retrospective cohort registry, with voluntary participation and no financial remuneration. All patients admitted for COVID-19 in one of the participating hospitals were suitable for the study, and researchers followed up until discharge or death. There were no exclusion criteria except for patients’ explicit refusal to participate. From 23 March 2020 to 20 April 2020, all patients fulfilling inclusion criteria from 23 centers in 19 cities and 4 countries (Ecuador, Germany, Italy and Spain) were assessed in the present manuscript.

### 2.2. Data Extraction

Epidemiological, clinical and outcome data were extracted from electronic medical records. Data were anonymized and stored electronically on a secure, password-protected system. Throat swab samples were obtained from all patients upon admission and tested using real-time reverse transcriptase–polymerase chain reaction assays according to the WHO recommendation. Additionally, data from routine hematological, biochemical and coagulation tests, as well as from chest x-ray and computed tomography reports, were extracted. Comorbidities were evaluated upon admission (including hypertension, dyslipidemia, diabetes mellitus, obesity, current smoking, renal failure, lung disease, cardiac disease, cerebrovascular disease, connective tissue disease, liver disease, cancer, Parkinson’s disease and dementia). All prescribed drugs upon admission were also recorded.

All-cause in-hospital mortality was the primary outcome. Other events were recorded, including invasive mechanical ventilation, non-invasive mechanical ventilation, respiratory insufficiency, heart failure, renal failure, bleeding, sepsis and embolic events. Complications during in-hospital stays were evaluated, including the following: respiratory failure, heart failure, renal failure, pneumonia, sepsis, systemic inflammatory response syndrome (SIRS), clinically relevant bleeding, hemoptysis and embolic events. Definitions of each event can be found at the registry’s website, although final allocation depended on local researchers’ criteria [15,16,17].

### 2.3. Other Definitions

During laboratory blood testing, levels of C-reactive protein (CRP), procalcitonin, ferritin, D-Dimer, lactate dehydrogenase, troponin, transaminases and triglycerides were defined as elevated according to local laboratory cutoff values. However, suggested values were given as follows: CRP ≥ 10 mg/L, procalcitonin ≥ 0.5 ng/dL, ferritin ≥ 335 ng/mL, D-Dimer ≥ 0.5 mg/dL, troponin (I or T) > 99th percentile, lactate dehydrogenase ≥ 250 U/L, transaminases (aspartate transaminase or glutamate transaminase) ≥ 40 U/L and triglycerides ≥ 200 mg/dL. Lymphopenia was defined as lymphocyte counts < 1000/mm^3^, and severe lymphopenia was defined as lymphocyte counts < 500/mm^3^.

### 2.4. Ethical Considerations

The study was performed according to the ethical principles of the Declaration of Helsinki and Good Clinical Practice Guidelines, and the research protocol was approved by the Ethics Research Committee from the Hospital Clínico San Carlos (Madrid, Spain) (20/241-E) and the Spanish Agency for Medicines and Health Products classification (EPA-0D). Written informed consent was waived due to the characteristics of the anonymized registry and the severity of the situation. However, verbal authorization from the patient (or familiar or caregiver when unavailable) was requested.

### 2.5. Statistical Analysis

We followed STROBE guidance for observational research [18]. Comparison between groups with or without ILDs was performed according to types of variables. On one hand, for quantitative variables, mean comparisons were conducted by using Student’s t-test if there was normality or the Mann–Whitney test otherwise. Normality was assessed with the Shapiro–Wilks test. On the other hand, comparison of percentages between groups was performed with Fisher’s exact test for dichotomous variables or a chi-square test for contingency tables with more than two categories and if cell count was higher than five.

Logistic regression was carried out to study factors associated with ventilatory support during admission or death. A stepwise approach with a backward–forward method was used to select variables in the multivariable model, and the Akaike information criterion [19] was used as the decision criterion. 

Software used for all analysis was R in its 4.0.2 version (R Core Team, 2022). *p*-values lower than 0.05 were considered statistically significant.

## 3. Results

From a cohort of 6894 patients admitted for COVID-19, 114 (1.6%) patients with a prior ILD diagnosis were identified [20] and compared with those without ILDs.

The mean (SD) age of ILD patients was 72.4 (13.6) years, and 65.8% were male. Comparing ILD vs non-ILD patients, we found differences in age (72.4 vs. 62.9; *p* < 0.001) and in a number of comorbidities: hypertension (66.7% vs. 48.1%; *p* < 0.001), dyslipidemia (50.9 vs. 29.9; *p* < 0.001), diabetes (34.2 vs. 18.1; *p* < 0.001), cardiovascular disease (43.9 vs. 20; *p* < 0.001), obesity (34.2 vs. 18.9; *p* < 0.001), chronic kidney disease (20.2 vs. 6.3; *p* < 0.001), cerebrovascular disease (14 vs. 7.4; *p* = 0.012), connective tissue disease (11.4 vs. 2.3; *p* < 0.001) and liver disease (9.6 vs. 3.6; *p* = 0.002). There were also significant differences in frequencies of home oxygen therapy (17.5 vs. 3; *p* = 0001) and respiratory failure on admission (49.1 vs. 32.3; *p* < 0.001). Regarding laboratory parameters, there were differences in the percentage of patients with elevated lactate dehydrogenase (LDH) (72.8 vs. 58.9; *p* = 0.004), elevated C-reactive proteins (88.6 vs. 79.4; *p* = 0.024) and elevated D-dimers (64.9 vs. 52.4; *p* < 0.011). Finally, differences were seen in acute kidney injuries during admission (28.1 vs. 14.4; *p* < 0.001), O2 use during admission (77.2 vs. 67.8; *p* = 0.034), death during hospitalization (31.6 vs. 18.3; *p* < 0.001) and death before follow-up (36.8 vs. 19.8; *p* < 0.001) (Table 1).

Causes of death during admission were analyzed to identify differences between patients with ILDs vs. those without. Death from respiratory causes was much more frequent in patients with ILDs (20% vs. 9.8%) (Table 2).

Within the group of patients with ILDs, a bivariate and multivariable logistic regression was performed to predict those characteristics of the patients that were related to the need for ventilatory support (high-flow nasal cannula or invasive or non-invasive mechanical ventilation) during admission. We found that chronic kidney disease (OR: 5.63 95% CI: 1.50–26.99; *p* 0.017) and the presence of respiratory failure on admission (OR: 11.21 95% CI: 2.85–75,24; *p* 0.002) were associated with the need for ventilatory support during hospitalization (Table 3).

Finally, we performed a bivariate and multivariable logistic regression to predict death in ILD patients admitted for COVID-19 (Table 4). We identified associations with the following variables: older age (OR: 1.09 95% CI: 1.04–1.15; *p* < 0.001), chronic kidney disease (OR: 6.22 95% CI: 1.48–31.39; *p* = 0.017), LDH elevation OR: 7.65 95% CI: 1.85–43.10; *p* = 0.010) and acute kidney failure during admission (OR: 4.20 95% CI: 1.30–14.41; *p* = 0.018).

Mean comparison was carried out using Student’s *t*-test in the presence of normality, and if the data was non-normal, the Mann–Whitney U test was used instead. For quality variables, the comparison of percentages between groups was carried out using Fisher’s exact test for dichotomous variables or a chi-square test for contingency tables with more than two categories if the cell count was higher than five.

A combined cause of death is defined as when several of the factors described previously caused the death of the patient, and it was not possible to identify one as the only one.

## 4. Discussion

In our multicenter international prospective study, ILD patients admitted for COVID-19 were older, had more comorbidities, required more ventilatory support during admission and had worse prognoses than non-ILD patients. As seen in other patients with COVID-19, renal failure, whether acute or chronic [21,22], contributes to more severe infections and even increases the risk of death in ILD patients. Moreover, older age and elevated LDH on admission were able to predict death in those patients.

ILD patients are particularly susceptible to respiratory infections. To begin with, they are patients who, in many cases, suffer from a functional limitation in their lungs. Further, it has been described that viral infections can trigger an exacerbation of ILDs [23,24], which would aggravate any established or in-development COVID-19-based pneumonia.

In our cohort, we saw that ILD patients have certain baseline aspects that differentiate them. For instance, patients with ILDs are older, as these diseases are associated with advanced age [25,26], such as idiopathic pulmonary fibrosis, the most frequent ILD [27]. We also report that ILD patients more frequently have comorbidities, as they are usually more complex patients, which also contributes to a worse prognosis; they also more frequently present elevated inflammatory reactants in blood or serum.

Many ILD patients suffer respiratory failure and are on home oxygen therapy, which contributes to the fact that they more frequently arrive at the hospital with respiratory insufficiency and require ventilatory support during hospitalization.

Finally, in these patients, we found independent determinants that predict a higher risk of death, such as the presence of acute or chronic renal failure, older age and elevated LDH. These factors should be flagged upon admission to diminish the risk of any fatal outcome. In addition, we report that ILD patients die more frequently from respiratory causes compared to non-ILD patients (Table 2).

As previously presented [28], patients suffering from any lung diseases had worse 30-day survival than those without a prior history of lung disease. A sub-analysis of each lung disease showed that COPD and ILD had the worse survival of these patients. In the current study, this was further confirmed for ILD patients, who presented specific features. Therefore, our results are consistent with other published cohort studies of patients with ILD: these patients have a more severe form of SARS-CoV-2 infection and COVID-19 as well as higher mortality [29].

We previously reported that patients with COVID-19 have elevated serum biomarkers involved in pulmonary fibrosis [30], and other studies have demonstrated the fibrotic sequelae that COVID-19 can leave behind [31], which could contribute to worsening the underlying disease in these patients who already have interstitial involvement prior to infection.

The strengths of our study are that this is an international multicenter study with a large sample size, and it represents real-world data. However, our study has several limitations to consider. First, we did not analyze individual types of ILDs, which may encompass a broad spectrum of diagnoses with different prognoses. Second, we did not take into account the immunosuppressive medication that each patient was receiving or the data from their pulmonary function tests, which are variables that could influence patient prognoses. Furthermore, the confidence intervals are wide, mainly due to the small sample size of ILD patients. In addition, this was a multicenter study conducted during the first wave of the COVID-19 pandemic, which may have contributed to missing data on key variables that were not collected and were not taken into account in the statistical analysis.

## 5. Conclusions

Patients with ILDs admitted for COVID-19 pneumonia were older and had more comorbidities than those without ILDs. During admission, these patients more frequently required ventilatory support and were more likely to have a fatal outcome, with the most likely cause of death being respiratory failure. It is paramount to recognize them upon admission, as they are at elevated risk for several complications and death.

## Figures and Tables

**Table 1:** Study patient characteristics.

	ILD Patients *n* = 114	Non-ILD Patients *n* = 6840	Total *n* = 6954	*p*-Value
Age, years	72.4 (13.6)	62.9 (18)	63.1 (18)	<0.001
Male sex	75 (65.8%)	4009 (58.6%)	83 (61.5%)	0.148
Current smoker	6 (5.3)	433 (6.3)	439 (6.3)	0.757
Comorbidities				
Hypertension	76 (66.7%)	3287 (48.1)	3363 (48.4)	<0.001
Dyslipidemia	58 (50.9%)	2044 (29.9%)	2102 (30.0%)	<0.001
Diabetes	39 (34.2%)	1237 (18.1%)	1276 (18.3%)	<0.001
Cardiovascular disease	50 (43.9%)	1367 (20%)	1417 (20.4%)	<0.001
Obesity	39 (34.2)	1293 (18.9)	1332 (19.2)	<0.001
Chronic kidney disease	23 (20.2)	430 (6.3)	453 (6.5)	<0.001
Cerebrovascular disease	16 (14.0)	503 (7.4)	519 (7.5)	0.012
Connective tissue disease	13 (11.4)	159 (2.3)	172 (2.5)	<0.001
Liver disease	11 (9.6)	247 (3.6)	258 (3.7)	0.002
Home oxygen therapy	20 (17.5)	203 (3)	223 (3.2)	<0.001
Respiratory insufficiency on admission	56 (49.1)	2210 (32.3)	2266 (32.6)	<0.001
† Laboratory findings				
Lymphocytes, ×10^9^/L	1373.5 (2542.1)	1401.8 (2616.2)	1401.3 (2614.8)	0.912
Elevated LDH	83 (72.8)	4027 (58.9)	4110 (59.1)	0.004
Elevated C-reactive proteins	101 (88.6)	5430 (79.4)	5531 (79.5)	0.024
Elevated D-dimers	74 (64.9)	3583 (52.4)	3657 (52.6)	0.011
Acute kidney failure during admission	32 (28.1)	984 (14.4)	984 (14.4)	<0.001
O_2_ used during admission	88 (77.2)	4636 (67.8)	4724 (67.9)	0.034
Death during hospitalization	36 (31.6%)	1252 (18.3%)	1288 (18.5%)	<0.001
Death before follow-up	42 (36.8%)	1351 (19.8%)	1393 (20.0%)	<0.001

Data are expressed as *n* (%) or mean (SD). ILD, interstitial lung disease; LDH, lactate dehydrogenase. † All laboratory findings are peak values except for lymphocytes, in which the lowest value is given.

**Table 2:** Causes of death.

	ILD Patients *n* = 114	Non-ILD Patients *n* = 6840	Total *n* = 6954	*p*-Value
Combined	5 (4.4%)	267 (3.9)	272 (3.9)	<0.001
Respiratory	23 (20.2%)	667 (9.8%)	690 (9.9%)	
Sepsis	3 (2.6%)	83 (1.2%)	86 (1.2%)	
SIRS	1 (0.9%)	45 (0.7%)	46 (0.7%)	
Renal Failure	2 (1.8%)	8 (0.1%)	10 (0.1%)	
Cardiovascular	0 (0%)	46 (0.7%)	46 (0.7%)	
Cerebrovascular	0 (0%)	22 (0.3%)	22 (0.3%)	
Other	0 (0%)	48 (0.7%)	48 (0.7%)	

ILD, interstitial lung disease.

**Table 3:** Bivariate and multivariable logistic regression to predict need for ventilatory support during admission.

	Ventilatory Support During Admission *		Bivariate Analysis		Multivariable Analysis	
	Yes	No	OR (95% CI)	*p*-Value	OR (95% CI)	*p*-Value
Male gender	30 (40)	45 (60)	1.28 (0.57–2.95)	0.55		
Age	74.3 (11.2)	71.2 (15)	1.02 (0.99–1.05)	0.254		
Hypertension	30 (40)	45 (60)	1.28 (0.57–2.95)	0.55		
Diabetes mellitus	13 (33.3)	26 (66.7)	0.80 (0.34–2.79)	0.587		
Obesity	14 (35.9)	25 (64.1)	0.87 (0.38–1.93)	0.732		
Smoker	1 (16.7)	5 (83.3)	0.31 (0.02–2.00)	0.29	0.26 (0.01–3.87)	0.36
Chronic kidney disease	13 (56.5)	10 (43.5)	2.60 (1.03–6.76)	0.045	5.63 (1.50–26.99)	0.017
Heart disease	20 (40)	30 (60)	1.16 (0.54–2.49)	0.704		
Connective tissue disease	2 (15.4)	11 (84.6)	0.26 (0.04–1.04)	0.092	0.27 (0.03–1.38)	0.149
Elevated PCR	39 (39)	61 (61)	1.44 (0.44–5.60)	0.567		
Elevated LDH	37 (45.1)	45 (54.9)	3.43 (1.34–10.02)	0.015	2.84 (0.83–11.23)	0.109
Lymphocyte count	1477.9 (3776.3)	1326.9 (1464.8)	1.00 (1.00–1.00)	0.77		
Respiratory insufficiency on admission	41 (51.2)	39 (48.8)	16.29 (4.51–104.90)	<0.001	11.21 (2.85–75.24)	0.002
Acute kidney failure during admission	17 (53.1)	15 (46.9)	1.28 (0.57–2.95)	0.04		

* Ventilatory support = high-flow nasal cannula or mechanical ventilation (invasive or non-invasive). Variable selection for multivariable model done according to the Akaike information criterion.

**Table 4:** Bivariate and multivariable logistic regression to predict death.

	Death During Admission *		Bivariate Analysis		Multivariable Analysis	
	Yes	No	OR (95% CI)	*p*-Value	OR (95% CI)	*p*-Value
Male gender	26 (34.7)	49 (65.3)	0.76 (0.34–0.1.70)	0.505	0.32 (0.10–0.97)	0.05
Age	78.2 (10.9)	69.3 (14)	1.07 (1.03–1.11)	0.001	1.09 (1.04–1.15)	<0.001
Hypertension	31 (40.8)	45 (59.2)	1.69 (0.74–4.02)	0.219		
Diabetes mellitus	14 (35.9)	25 (64.1)	0.97 (0.42–2.17)	0.94		
Obesity			0.02 (−0.14–0.19)	0.767	0.08 (−0.06–0.22)	0.241
Smoker	3 (50)	3 (50)	1.63 (0.29–9.21)	0.561	3.40 (0.29–41.99)	0.326
Chronic kidney disease	15 (65.2)	8 (34.8)	4.44 (1.73–12.22)	0.003	6.22 (1.48–31.39)	0.017
Heart disease	23 (46.0)	27 (54.0)	2.02 (0.94–4.41)	0.075		
Connective tissue disease	4 (30.8)	9 (69.2)	0.74 (0.19–2.43)			
Elevated PCR	38 (37.6)	63 (62.4)	0.10 (−0.15–0.34)	0.429	1.36 (0.41–5.29)	0.631
Elevated LDH	35 (42.2)	48 (57.8)	2.50 (1.01–6.87)	0.058	7.65 (1.85–43.10)	0.01
Lymphocyte count	1222.7 (1751.2)	1453.2 (2882.5)	1.00 (1.00–1.00)	0.663		
Respiratory insufficiency on admission	35 (43.8)	45 (56.2)	2.89 (1.17–7.92)	0.028		
Acute kidney failure during admission	20 (62.5)	12 (37.5)	4.47 (1.91–10.91)	0.001	4.20 (1.30–14.41)	0.018
Thromboembolic event	3 (60.0)	2 (40.0)	2.65 (0.42–20.80)	0.296		

* Mean (SD) or *n* (%) as appropriate. LDH, lactate dehydrogenase. Variable selection for multivariable model done according to the Akaike information criterion.

## Data Availability

Not applicable.
